# Etherization Followed by Death

**Published:** 1864-04

**Authors:** 


Etherization followed by Death.—At the meeting of the Imperial Society of Medicine in Lyons, on July 20, M. Chassagny communicated the case of a lady, aged 40, to whom ether was administered previously to the removal of an urethral polypus and two sebaceous cystic tumors on the head. Thirty *grammes* of ether (rather less than an apothecary's ounce) were used; but the anæsthesia produced was incomplete, and the patient was aware that the operations were being performed. The administration of the anæsthetic was not pushed further, because the stage of excitement did not manifest itself, and because, on the contrary, general coldness and slowness of the pulse were present. On the completion of the operation, which occupied a quarter of an hour, vomiting set in; the coldness increased, and was accompanied by clammy sweats; and the patient had convulsions, attended with foaming at the mouth. The attack passsd away in a few moments, but soon returned with equal intensity. After the fourth attack, the patient died. M. Chassagny considered that the patient had died of eclampsia induced by etherization, which was thus the indirect cause of death. She had previously been subject to epileptic vertigo.—*British Med. Journ.*, from *Gaz. Med. de Lyon*, 16 October, 1864.

